# Home-based exercise improves quality of life in breast and prostate cancer survivors: A meta-analysis

**DOI:** 10.1371/journal.pone.0284427

**Published:** 2023-04-20

**Authors:** Lauren C. Bates-Fraser, Sasha Riley, Cameron Stopforth, Kaileigh Moertl, Kyle Edgar, Lee Stoner, Erik D. Hanson

**Affiliations:** 1 Department of Exercise and Sport Science, University of North Carolina at Chapel Hill, Chapel Hill, NC, United States of America; 2 Human Movement Science Curriculum, University of North Carolina at Chapel Hill, Chapel Hill, NC, United States of America; 3 Lineberger Comprehensive Cancer Center, Chapel Hill, NC, United States of America; University of Toronto Temerty Faculty of Medicine, CANADA

## Abstract

**Background:**

Breast (BCa) and prostate (PCa) cancer are two of the most common but survivable cancers. One important component of survivorship that is impacted by treatment long term is diminished quality of life (QoL). Supervised exercise improves QoL and subsequent outcomes but is not accessible for all survivors. Additionally, many factors influence QoL including physical activity (PA), cardiorespiratory fitness (CRF), physical function, and fatigue. However, the COVID-19 pandemic has highlighted the need to increase access to exercise beyond supervised exercise facilities. Home-based exercise may provide a feasible alternative for cancer survivors especially for those living in rural communities.

**Objectives:**

The primary aim is to investigate the effects of home-based exercise training (Pre-training vs. Post-training) on QoL in BCa/PCa. A secondary aim is to investigate PA, CRF, physical function, and fatigue and potential moderators (age, cancer-type, intervention duration and type). Home-based exercise trials (randomized crossover or quasi-experimental design) with adults (aged 18 years and over) breast or prostate cancer survivors (not currently undergoing chemotherapy or radiation treatment) were eligible for inclusion.

**Data sources:**

Electronic databases were searched (inception-December 2022) for studies which included adult BCa or PCa survivors (not currently on chemotherapy/radiation), at least measured QoL, and undergoing unsupervised, home-based exercise training.

**Appraisal and synthesis methods:**

Initially, 819 studies were identified, from which 17 studies (20 effects) involving 692 participants were extracted. Effect sizes were calculated as standardized mean differences (SMD). Data were pooled using a 3-level model with restricted maximum likelihood estimation. Pooled SMD was used to assess the magnitude of effect, where <0.2, 0.2, 0.5, and 0.8 was defined as trivial, small, moderate, and large respectively.

**Results:**

Home-based exercise resulted in small improvements in QoL (SMD = 0.30, 95% CI 0.01, 0.60, p = 0.042), PA (SMD = 0.49, 95% CI 0.26, 0.75, p<0.001) and CRF (SMD = 0.45, 95% CI -0.01, 0.91, p = 0.056). Physical function (SMD = 0.00, 95% CI -0.21, 0.21, p = 1.000) and fatigue (SMD = -0.61, 95%CI -1.53, 0.32, p = 0.198) did not change.

**Conclusions:**

Home-based exercise results in small improves QoL in BCa/PCa survivors, independent of cancer type, intervention duration and type, or age. Home-based exercise also improves PA and CRF enhancing survivorship. Therefore, home-based exercise is an efficacious alternative option to improve QoL for BCa and PCa survivors especially for those who live in rural communities or lack access to exercise facilities.

## 1. Introduction

The survival rates for breast (BCa) and prostate (PCa), the two most common non-dermatological cancers, are high at 98 and 90% [1], respectively. However, of the estimated 7.5 million BCa and PCa survivors [2], many are burdened by diminished quality of life (QoL). Evidence demonstrates that declines in QoL [3–8] can be attributed to the cancer- and cancer treatment- related side effects [9–11], including increased fatigue [11,12] and subsequent reductions in physical activity [13,14] (PA), cardiorespiratory function [15] (CRF), and physical function [16]. One approach to combat reduced QoL is supervised exercise training. Further, it is known that supervised exercise decreases fatigue [11,12], can potentially increase PA [13,14], CRF [15] and physical function [16], and improves QoL [5–8]. However, supervised exercise is not always feasible due to many challenges at the environmental and behavioral levels [17–19]. Commonly reported barriers to exercise engagement include lack of access to exercise facilities, time to commute to exercise facilities around work or treatment schedules, health disparities (lack of resources in low socioeconomic areas) and residing in a rural community [17–19]. The importance of these barriers have become particularly emphasized in the wake of the COVID-19 pandemic [20]. Alternatively, home-based, unsupervised exercise may minimize the likelihood of the aforementioned barriers impeding exercise initiation and adherence in BCa and Pca survivors. However, the effect of home-based unsupervised exercise on QoL and other outcomes such as PA, CRF, physical function, and fatigue remain unknown.

The strongest form of evidence for guiding policy development, including exercise prescription, are meta-analytical findings [21,22]. While previous meta-analyses do support the use of supervised exercise for improving QoL in BCa and PCa survivors [23–25], such support is unavailable for unsupervised exercise. Home-based unsupervised exercise is a potentially advantageous strategy for BCa/PCa survivors who live in rural communities or lack access to supervised training. There are several important considerations in conducting such a meta-analysis, including the need to account for effect size dependencies and potential effect moderators. With respect to the former consideration, a range of instruments have been used to quantify QoL, including FACT-B [26], FACT-G [27], FACT-P [28], SF-36 [29], IBCSG [30], EORTC [31], PORPUS [32], PFS [33]. Some studies have used more than one of these tools, meaning that there is a need to account for multiple effects nested within a given study. No standardized exercise prescription exists for BCa/PCa survivors leading to studies with a variety of exercise training durations and types of exercise further complicating between study comparisons. Adjustments can be made for the nested effects using 3-level meta-analysis, which calculates the overall effect size while accounting for variance between studies (Level 3 tau^2^), variance between effect sizes extracted from the same study (Level 2 tau^2^), and sampling variance (Level 1 tau^2^) [34]. The 3-level model can also assess whether the QoL instruments moderates the effect, and thereby account for both level 2 and level 3 variance, and test additional moderators that may help to explain level 3 variance. Important potential moderators include the duration of the intervention and the form of exercise prescriptions (frequency, intensity, time, and type). Examination of the control groups were outside the scope of this meta-analysis. However, taking a 3-level meta-analytic approach will allow us to consolidate the existing literature most effectively to guide exercise recommendations for BCa/PCa survivors. Furthermore, in a 3-level model we can also account for variance due to potential moderators such as age or cancer type.

Finally, current American College of Sports Medicine Guidelines recommend engaging in 150 minutes of moderate-to-vigorous aerobic physical activity and resistance training at least 2 days per week [35,36]. Therefore, when comparing the effects of home-based unsupervised exercise, consideration for the exercise prescription is also key to inform subsequent recommendations and current practice. We must consider the type (i.e., aerobic, resistance training, or combined), frequency (i.e., days per week), and duration of the exercise intervention (i.e., number of weeks) when comparing the effect of exercise in BCa/PCa survivors.

### 1.1 Objective

The primary objective of this study was to examine the effect of home-based exercise on QoL in BCa/PCa survivors. Secondary objectives were to examine the effect of home-based exercise on PA, CRF, physical function, and fatigue. Additionally, exploratory analyses were conducted to examine potential differences in cancer type (BCa vs. PCa), study duration (≤ 12 weeks or ≥ 13 weeks), age (≤ 59 years old or ≥ 60 years old), exercise type (aerobic, resistance training, combined, or other), and assessment type.

## 2. Methods

This meta-analysis was carried out in accordance with PRISMA (Preferred Reporting Items for Systematic Reviews and Meta-Analyses) guidelines and registered with PROSPERO.

### 2.1 Data sources and searches

Electronic databases (PubMed, Embase, Scopus, SPORTDiscus, and Google Scholar) were searched by two authors (LB, KM) utilizing the search: (prostate cancer OR prostate tumor OR prostate neoplasm OR prostate malignancy OR breast cancer OR breast tumor OR breast malignancy OR hormonal cancer) AND (quality of life OR functional movement OR activities of daily living OR timed up and go OR 6 minute walk test OR gait OR balance OR physical function OR physical activity OR fatigue) AND (home based exercise OR home exercise training OR exercise at home OR unsupervised exercise OR home exercise OR walking). The reference lists of all identified trials and relevant reviews or editorials were also examined. The search was limited to English language studies published between inception and December 2022.

### 2.2 Article selection

For the purpose of this meta-analysis, the term ‘article’ is used synonymously with ‘study’, and ‘trial’ is the unit included in the meta-analysis. A given article may have resulted in more than one eligible ‘trial’ if the article included more than one intervention group. Initially, article titles and abstracts were screened for relevance. The full-text of potentially eligible articles were obtained to review eligibility for inclusion. The following criteria were used to select trials for inclusion in the review: i) Participants Inclusion: Adults (aged 18 years and over), ii) cancer survivors (i.e., not currently undergoing chemotherapy or radiation treatment), iii) breast or prostate cancer survivors (we will define survivorship as post-surgery and/or no longer undergoing chemotherapy or radiation treatment) and exclusion: i) Under the age of 18, ii) supervised exercise training and/or group-based exercise training, iii) cancer patient currently undergoing chemotherapy or radiation treatment.

In trials with multiple treatment arms and a single control group, the sample size of the control group was divided by the number of treatment groups to avoid over-inflation of the sample size. Repeated publications for the same studies were excluded. Two researchers (LB, KM) completed the study selection independently.

### 2.3 Data extraction and quality assessment

Data extracted for each eligible trial included bibliographic information (author, publication year), baseline participant characteristics, details of intervention(s), and results of reported outcomes. Study quality was assessed using the NIH Study Quality Assessment Tool [37] and RoB2: revised Cochrane risk-of-bias tool for randomized trials (range 1–3), which includes items related to randomization, deviations from intended interventions, missing data, measurement of the outcome, selection of the reported result, and overall bias [38]. Because it is difficult (if not impossible) to blind participants to an exercise intervention, we considered the blinding of the operator to the outcome assessment as a quality criterion. Data extraction, quality assessment, and scrutiny of the exercise interventions were completed by two authors (LB, SR).

### 2.4 Data synthesis

For each outcome of interest, the pre- and post-intervention values (mean and standard deviation) were entered into a spreadsheet. When mean differences and associated standard deviations were not published, they were estimated from the pre- and post-intervention values based on methods from the Cochrane Handbook for Systematic Reviews of Interventions. For studies reporting multiple time points, only the final time point was used in analyses. Primary study outcomes were QoL, PA, CRF (VO_2_), physical function, and fatigue variables. Two authors independently gathered data by reviewing literature, and then results were compared (LB and SR or KE or CS). Aggregation and calculation of final results was conducted by one author (LS).

### 2.5 Data analysis

A single author (LS) conducted statistical analysis using the metafor-package [39] for the R statistical environment (RKWard version 0.7.1). Considering the QoL measures are expressed using different units and scales, standardized mean differences (SMD) were calculated. Data were pooled using a 3-level model with restricted maximum likelihood estimation [40,41]. The pooled SMD was used to assess the magnitude of effect, where <0.2, 0.2, 0.5, and 0.8 was defined as trivial, small, moderate, and large respectively [42]. A 3-level meta-analysis model was used to account for effect size dependency when a given study reported more than one effect size (i.e., multiple QoL measures) [34]. These models permit calculation of a summary (or average/overall) effect size (SMD) estimate while accounting for variance between studies (Level 3 tau^2^), variance between effect sizes extracted from the same study (Level 2 tau^2^), and sampling variance (Level 1 tau^2^). Subsequent to running the 3-level models, we examined the robustness of the pooled results, the potential for publication bias, and heterogeneity. In the event of significant heterogeneity, moderator analysis was conducted. The α level was set *a priori* at α = 0.05 for the pooled SMD estimate and α = 0.10 for moderator analysis.

Robustness was examined at each level of the model using Studentized residuals and Cook’s distances. Studies with a studentized residual larger than the 100 x (1–0.05/(2 X k)) were considered potential outliers. Studies with a Cook’s distance larger than the median plus six times the interquartile range of the Cook’s distances were considered to be influential. Publication bias was qualitatively examined through inspection of Begg’s funnel plot, and funnel plot asymmetry was quantitively tested using the regression intercept test, with the standard error of the observed outcomes specified as a moderator in the 3-level model [43]. Last, heterogeneity was assessed the Q-test [44] and the I^2^ statistic [45], where <25%, 25%-75%, >75% indicate low, moderate and considerate risk, respectively. Significant heterogeneity indicated that effect sizes could not be treated as estimates of one common effect size. The level of variance (tau^2^) was examined using log-likelihood ratio tests, where the full model was compared to separate models excluding level-2 (within-study, e.g., multiple QoL outcomes) and level-3 (between-study) variance parameters. Subsequently, the model was extended with *a priori* determined moderator analysis (cancer type, assessment type, age, exercise duration, or exercise type). The amount of variance explained through the specific of moderator(s) was examined by calculating the percentage reduction in the tau^2^ at each level of the model. In the event of a significant moderator, subgroup analysis was performed through use of dichotomous dummy coded variables.

## 3. Results

### 3.1 Literature search and selection

**Fig 1:**
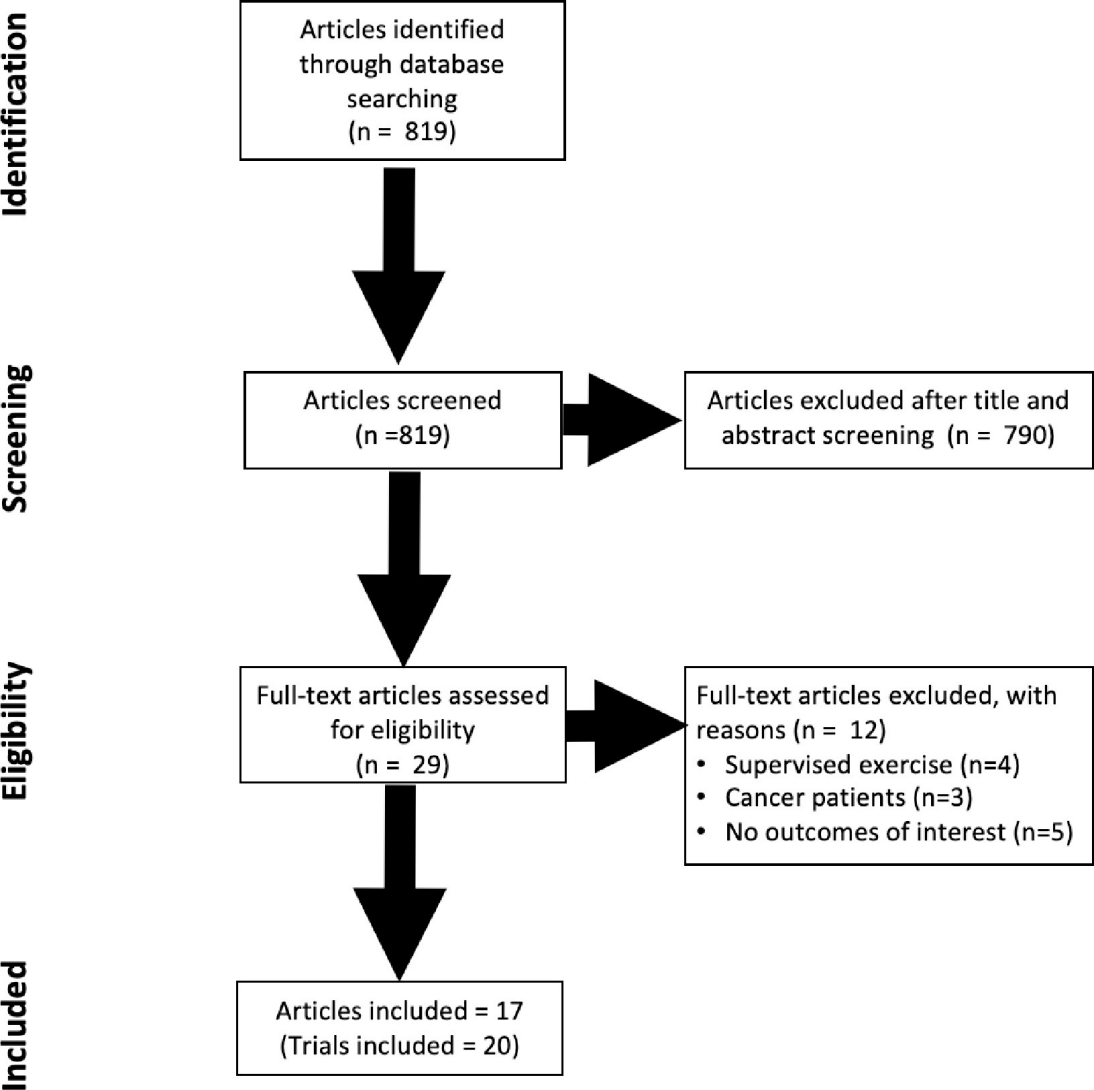
Flow diagram of study selection: Identification, screening, eligibility, and inclusion.

**Fig 1** outlines the literature search strategy. A total of 949 potentially eligible articles were identified. Following screening of titles and abstracts, 914 articles were excluded because they did not meet selection criteria. Of these, 35 randomized cross-over trials underwent full text review and 18 were excluded due to i) supervised exercise component [46–51], ii) participants undergoing cancer treatment [52–56], iii) or outcome of interest [57–63]. The final analysis included 17 studies (20 trials) [64–80] with 17 studies reporting QoL, 10 PA, 6 CRF, 7 physical function, and 14 fatigue.

### 3.2 Description of the included studies

#### 3.2.1 Study setting and participants

Included trial characteristics are summarized in Table 1. The trials were carried out in the US (n = 9), Canada (n = 4), Italy (n = 1), Turkey (n = 1), Netherlands (n = 1), UK (n = 1), India (n = 1), Germany (n = 1), and Denmark (n = 1). The number of participants in each trial ranged from 7 to 249 (median = 20, mean = 28.6). Fourteen trials included only BCa survivors and five trials only PCa survivors. The mean age of the participants ranged from 51 to 72 years (60 (7)). Only thirteen trials reported baseline body mass index (kg/m^2^). The mean body mass index was 28.8 (1.4).

**Table 1:** Summary of studies included in meta-analysis.

Authors	Quality	Country	Cancer Type	Average time since treatment	Cancer Stage	BMI	Sample [n (total/F); age (y, mean (SD or range)]	Exercise	Duration (weeks)/ Frequency (days/week)	QoL	PA	CRF	Fatigue
Alibhai	3	Canada	Prostate	3 months ADT	Any, 53% stage I-II	29.6 (4.7)	[18 (0); 69.6 (8.1)]	AET/RT/flexibility	26/4-5	FACT-P/G	GLTEQ/ACC	VO2peak	FACT-F
Bali	2	USA	Breast	-	Any, 36% stage I	30.9 (7.25)	[44 (44); 60.5 (9.4)]	Gardening	52/NR	SF36	GLTEQ/ACC	2-min step test	-
Baruth	2	USA	Breast	<12 months	Any, 50% stage II	29.9 (6.9)	[20 (20); 54.9 (6.5))]	Walking	12/3-5	SF36/IBCSG	CHAMPS	-	FACT-F
Corrado	1	Italy	Breast	1–3 months	-	-	[30 (30); 57.8]	UB RT	2/7	EORTC	-	-	BFI
Culos-Reed	2	Canada	Prostate	28.5 months	Any	29.17 (5.24)	[31 (0); 64.8 (9.8)]	Walking/RT/stretching	12/3-5	EORTC	GLTEQ	6-min walk test	FSS
Ergun	1	Turkey	Breast	60.6 months	-	28.64 (5.15)	[20 (20); 55.05 (6.85)]	Walking	12/3	EORTC	-	-	BFI
Golsteijn	2	Netherlands	Prostate	186 months	-	26.39 (3.38)	[249 (37); 66.55 (7.07)]	Walking	16/NR	EORTC	SQUASH/ACC	-	CIS
Lahart	2	UK	Breast	<2 years	Stage I-III	27.25 (4.69)	[40 (40); 52.4 (10.3)]	AET	24/3-7	FACT-B	IPAQ	-	-
Melam	1	India	Breast	-	Stage I-II	-	[30 (30); 56 (3.5)]	UB RT	6/NR	EORTC	-	-	VAS
Santa Mina-A	2	Canada	Prostate	ADT	Any, 38% stage II	29.1 (3.4)	[32 (0); 72.1 (8.9)]	AET	24/3-5	FACT-P/PORPUS	GLTEQ	VO2peak	FACT-F
Santa Mina-B	2	Canada	Prostate	ADT	Any, 32% stage II	29.0 (4.0)	[34 (0); 70.6 (9.5)]	RT	24/3-5	FACT-P/PORPUS	GLTEQ	VO2peak	FACT-F
Mulero-Portela	2	Puerto Rico	Breast	<5 years	Any, 38% stage II	28.0 (7.0)	[13 (13); 51.2 (7.3)]	RT/AET	26/5	FACT-B	-	12-min walk	-
Nyrop	2	USA	Breast	2.7 years	Any, 34% stage II	-	[31 (31); 63.3 (6.9)]	Walking	6/NR	FACT-G	self-reported walking	-	VAS
Spahn	3	Germany	Breast	52.9 months	Any, 57% stage II	26.6 (4.1)	[25 (25); 55.3 (11.4)]	Walking	10/3	EORTC	-	-	VAS
Spector	2	USA	Breast	<24 months	Any, 46% stage II	30.2 (5.1)	[13 (13); 51.6 (12.9)]	AET/RT	16/2-4	FACT-B	IPAQ/ACC	VO2peak	FACIT-F
Stan-A	3	USA	Breast	>2 months	Stage 0-II, 44% stage I	Stage 0-II, 67% stage I	[6 (16); 63 (9.3)]	RT	12/3-5	FACT-B	-	-	MFSI-SF
Stan-B	3	USA	Breast	>2 months		-	[18 (18); 61.4 (7)]	Yoga	12/3-5	FACT-B	-	-	MFSI-SF
Villumsen	3	Denmark	Prostate	>3 months ADT	Any, 52% stage T2c-T3c	29.8 (0.6)	[23 (0); 67.6 (4.6)]	Exergaming	12/3	EORTC	GLTEQ	6-min walk test	FACT-F
Yuen-A	2	USA	Breast	<35 months	-	-	[7 (7); 53.1 (13.5)]	AET	12/3	PFS	-	6-min walk test	PFS
Yuen-B	2	USA	Breast	<35 months	-	-	[8 (8); 53.7 (11.3)]	RT	12/3	PFS	-	6-min walk test	PFS

Missing data indicated with

Abbreviations: BMI: Body mass index; SD: Standard deviation; QoL; quality of life; PA: Physical activity; CRF: Cardiorespiratory fitness; ADT: Androgen deprivation therapy; AET: Aerobic exercise training; RT: Resistance training; UB RT: Upper body resistance training; ACC: Accelerometry; NR: Not Reported.

#### 3.2.2 Interventions

A brief description of the exercise interventions is given in Table 1. The duration of interventions varied in length from 2 weeks to 52 weeks, with a median of 12 weeks. The trials included aerobic exercise only, resistance exercise only, aerobic and resistance training combined, walking, or other (yoga, exergaming, or gardening).

#### 3.2.3 Methodological quality assessment

The methodological assessment of included trials is summarized in Table 1. The quality of the included studies ranged from 1–3 (1 being poor and 3 being high quality), with a median quality score of 2 and a mean score of 2.1. Fourteen of the studies were randomized but only six stated that the allocation process was concealed, and outcome assessors were blinded. Ten studies adequately described withdrawal rates and reason for withdrawal, whereas only eight studies described adherence and two mentioned compliance to the intervention. Five studies were given a score of 3 and considered high quality because they reported a randomized design, measured PA at the beginning and end of study, included adherence/compliance/drop-out data, and the exercise prescribed met ACSM guidelines. Twelve studies were given a score of 2 and considered fair quality and three studies were given a score of 1 and considered poor quality.

### 3.3 Synthesis of results

#### 3.3.1 Quality of life

The pooled SMD was used to assess the magnitude of effect, where <0.2, 0.2, 0.5, and 0.8 was defined as trivial, small, moderate, and large respectively [42]. The pooled SMD effect from the 3-level model is presented initially, and then discussion of heterogeneity and effect measure modification follow.

In total *k* = 17 studies were included, from which *u* = 23 effect sizes were extracted with a total sample of n = 657. The adjudicated mean quality of the articles was 2 (out of 3), with a range of 1 to 3. The observed SMDs ranged from -1.1 to 1.9, with the majority of estimates (74%) being positive/beneficial.

**Fig 2:**
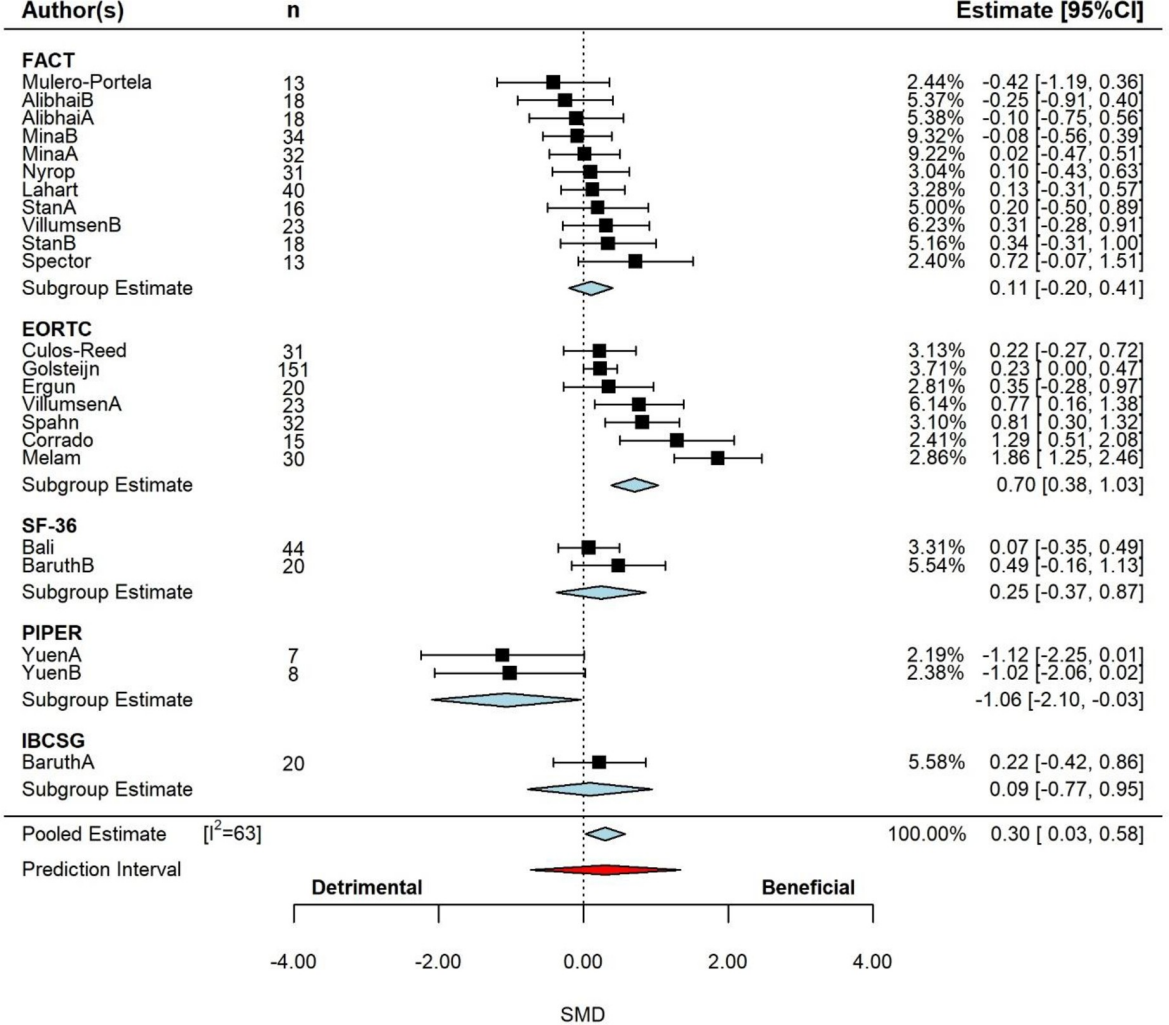
Pooled effect of exercise intervention on quality of life, with sub-group analysis by assessment type. (k = 17, u = 23, n = 508). Based on the 3-level model, there was a small but significant effect (SMD = 0.30, 95% CI: 0.01 to 0.60, p *=* 0.042). *Abbreviations*: CI, confidence interval; SMD, standardized mean differences.

The pooled SMD effect is presented in **Fig 2,** which contained the significant effect moderator assessment type. Data are presented by moderator. Based on the 3-level model, there was a small but significant effect (SMD = 0.30, 95% CI: 0.01 to 0.60, p *=* 0.042). Examination of the studentized residuals and Cook’s distance revealed that one study [71] may be an outlier and could be considered influential. Removal of Malem [71] decreased the size of the estimated effect, which remained small and significant (SMD = 0.21, 95% CI: 0.02 to 0.40, p *=* 0.030). No asymmetry (p = 0.174) was revealed following inspection of the funnel plot and regression test.

According to the Q-test, the true outcomes appear to be heterogeneous (Q = 65, p<0.001, tau^2^ = 0.26, I^2^ = 63%). The majority of the variance was at level-3 (77%), with the remainder of the variance at level-1 (23%). Neither cancer type (p = 0.483), study duration (p = 0.148), participant age (p = 0.279) or exercise prescription type (p = 0.795) were significant effect moderators. However, QoL measurement method was a significant moderator (p = 0.002), decreasing the level-3 variance by 52% and the overall variance by 40%. The subgroup analysis for QoL method is presented in **Fig 2**. There was a moderate significant increase (beneficial) for EORTC (p = <0.001), trivial to small non-significant increases for FACT (p = 0.498), SF-36 (p = 0.434) and IBCSG (p = 0.834), and a significant large decrease (beneficial) for PIPER (p = 0.044). The Malem study, which was identified as potential outlier/influential, utilized the EORTC method. With the removal of this study the effect for the EORTC method was reduced to small-moderate effect, but remained significant (SMD = 0.48, 95% CI: 0.24 to 0.73, p<0.001).

#### 3.3.2 Physical activity

In total of *k* = 10 studies were included, from which *u* = 14 effect sizes were extracted with a total sample of n = 630. The adjudicated mean quality of the articles was 2 (out of 3), with a range of 2 to 3. The observed SMD ranged from -0.2 to 1.7, with the majority of estimates being positive/beneficial (86%).

**Fig 3:**
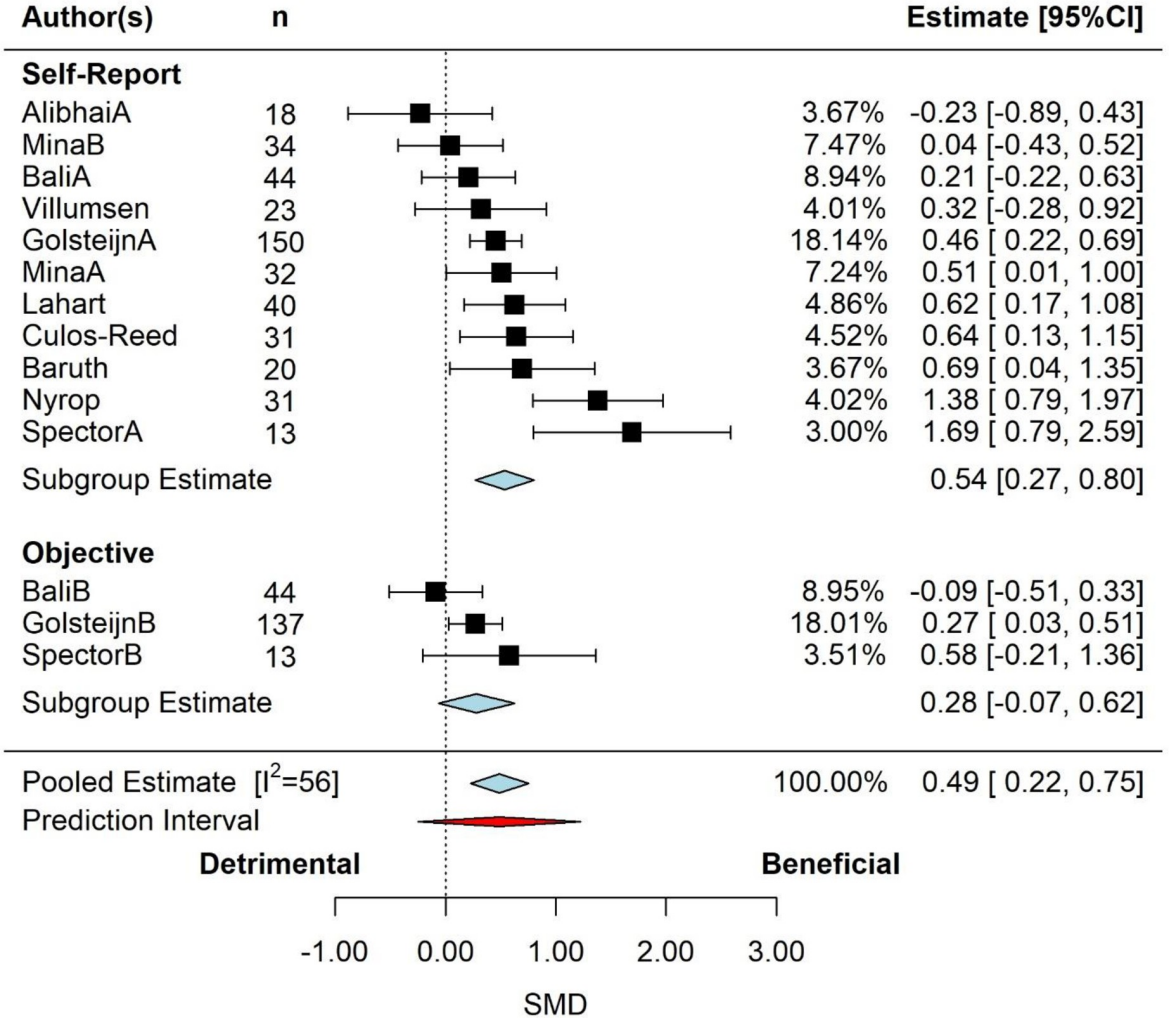
Pooled effect of exercise intervention on incidental physical activity, with sub-group analysis by assessment type (k = 10, u = 14, n = 630). Based on the 3-level model, there was a small but significant effect (SMD = 0.49, 95% CI: 0.22 to 0.75, p<0.001). *Abbreviations*: CI, confidence interval; SMD, standardized mean differences.

The pooled effect is reported in in **Fig 3** by moderator assessment type. Based on the 3-level model, there was a small but significant effect (SMD = 0.49, 95% CI: 0.22 to 0.75, p<0.001). Examination of the studentized residuals and Cook’s distance revealed no outliers or potentially influential studies or effects. Inspection of the funnel plot nor the regression test indicated asymmetry (p = 1.000).

According to the Q-test, the true outcomes appear to be moderately heterogeneous (Q = 34, p = 0.001, tau^2^ = 0.11, I^2^ = 56%). The majority of the variance was at level-3 (66%), followed by level-1 (28%) and level-2 (6%). Neither cancer type (p = 0.133), study duration (p = 0.109), participant age (p = 0.130) or exercise prescription type (p = 0.245) were significant effect moderators. However, PA measurement method was a significant moderator (p = 0.069), decreasing the level-3 variance by 5%, and the overall variance by 9%. The subgroup analysis for PA measurement method is presented in **Fig 3**. There was a moderate (SMD = 0.54) increase (beneficial) for self-reported PA (p<0.001), and a small (SMD = 0.28), non-significant increase for objectively measured PA (p = 0.113). With the removal of Nyrop [73] and Spector [75] (due to the potential of these studies being outliers) the pooled effect size was reduced for both self-reported PA (SMD = 0.38, 95% CI: 0.22 to 0.55, p<0.001) and for objectively measured PA (SMD = 0.16, 95% CI: -0.10 to 0.23, p = 0.230).

#### 3.3.3 Cardiorespiratory fitness

In total of *k* = 7 studies were included, from which *u* = 14 effect sizes were extracted with a total sample of n = 312. The adjudicated mean quality of the articles was 2 (out of 3), with a range of 2 to 3. The observed SMD ranged from -0.2 to 1.9, with the majority of estimates being positive/beneficial (86%).

**Fig 4:**
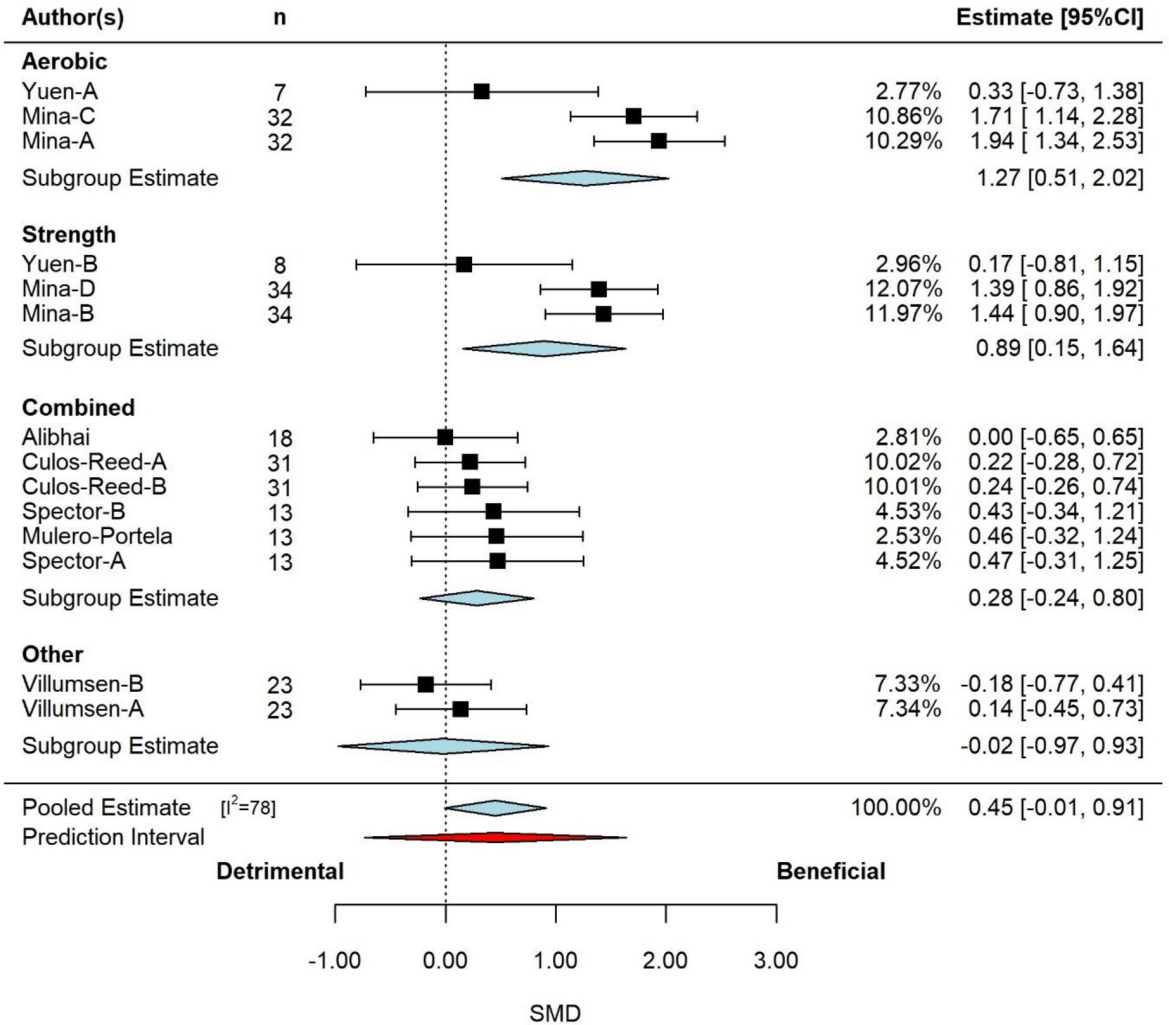
Pooled effect of exercise intervention on cardiorespiratory fitness, with sub-group analysis by exercise intervention type (k = 7, u = 14, n = 312). Based on the 3-level model, there was a small effect (SMD = 0.45, 95% CI: -0.01 to 0.91) which approached significance (*p =* 0.056). *Abbreviations*: CI, confidence interval; SMD, standardized mean differences.

The pooled effect is reported in in **Fig 4** on moderator exercise type. Based on the 3-level model, there was a small effect (SMD = 0.45, 95% CI: -0.01 to 0.91) which approached significance (*p =* 0.056). Examination of the studentized residuals and Cook’s distance revealed that one study (Santa Mina) may be an outlier and could be considered influential. Removal of Santa Mina due to the chance of it being an outlier decreased the size of the estimated effect (SMD = 0.20, 95% CI: -0.01 to 0.40, p *=* 0.067). Inspection of the funnel plot and the regression test indicated likely asymmetry (p = 0.067).

According to the Q-test, the true outcomes appear to be considerably heterogeneous (Q = 68, p<0.001, tau^2^ = 0.44, I^2^ = 78). The majority of the variance was at level-3 (75%), with the remainder of the variance at level-1 (25%). Neither measurement type (p = 0.212), cancer type (p = 0.852), study duration (p = 0.213), participant age (p = 0.852) or VO_2max_ unit expression (p = 0.403) were significant effect moderators. However, exercise prescription modality was approached being a significant moderator (p = 0.101), decreasing the level-3 variance by 39% and the overall variance by 29%. The subgroup analysis for exercise prescription modality is presented in **Fig 4**. There was a large, significant increase (beneficial) for aerobic (p = 0.001) or strength (p = 0.018) exercise prescription, a small, non-significant increase for combined (p = 0.286), and a trivial, non-significant decrease for other (p = 0.966). The Santa Mina [72] study included both aerobic and strength arms. Removal of this study decreased the pooled effect size for both aerobic (SMD = 0.33, 95%CI: -0.73 to 1.38, p = 0.541) and strength (SMD = 0.17, 95%CI: -0.81 to 1.15, p = 0.736), and combined became significant (SMD = 0.27, 95%CI: 0.01 to 0.53, p = 0.039).

#### 3.3.4 Physical function

In total of *k* = 7 studies were included, from which *u* = 12 effect sizes were extracted with a total sample of n = 285. The adjudicated mean quality of the articles was 2 (out of 3), with a range of 2 to 3. The observed SMD ranged from -0.6 to 0.5. An increase in 6-minute walk, 1-minute chair stands, 30 second chair stands, grip strength, and questionnaires (FACT) indicate a beneficial change. A decrease in timed-up-and-go also indicates a positive/beneficial change.

**Fig 5:**
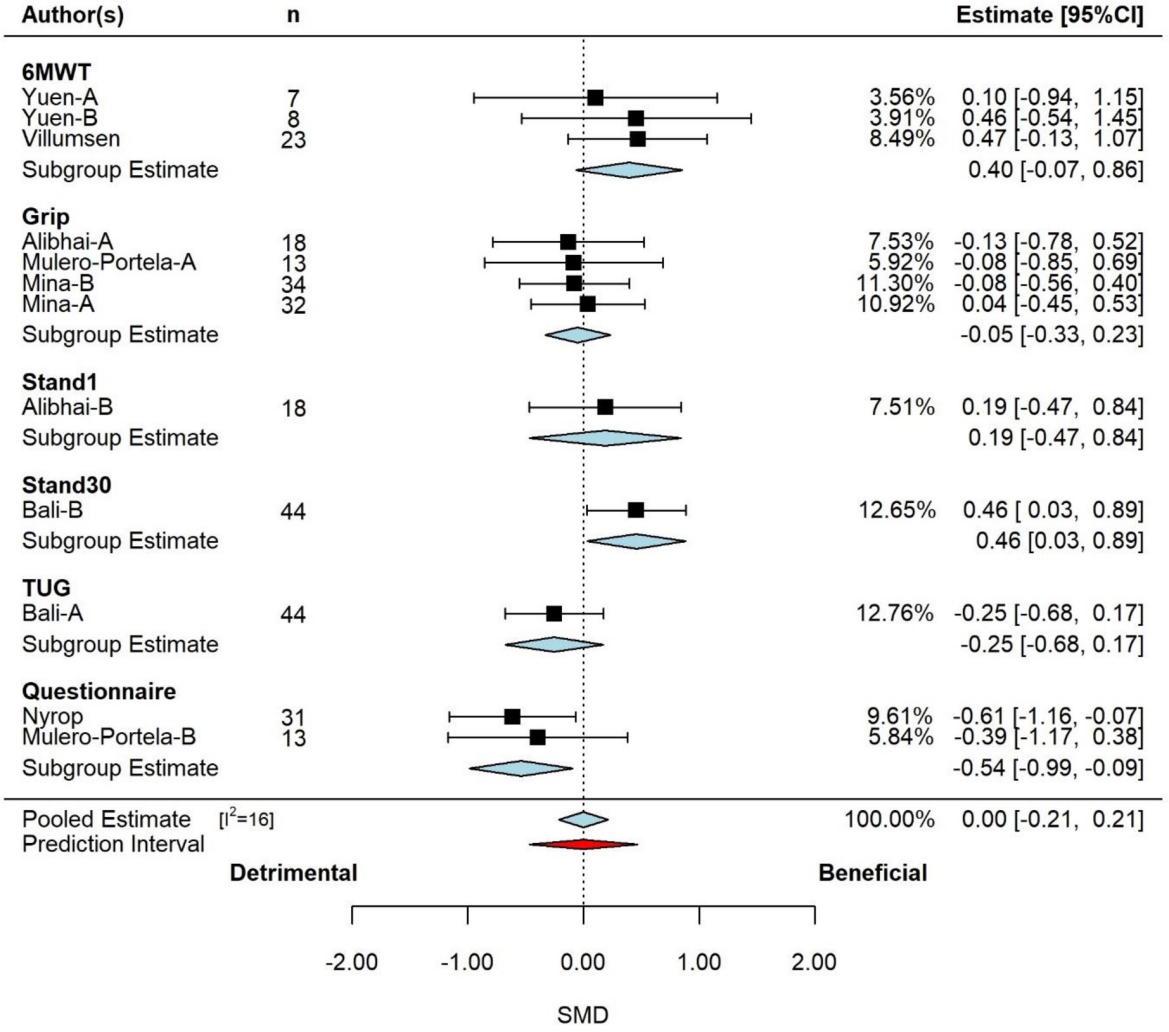
Pooled effect of exercise intervention on physical function, with sub-group analysis by assessment type (k = 10, u = 14, n = 508). Based on the 3-level model, there was no discernable effect (SMD = 0.00, 95% CI: -0.21 to 0.21, p = 1.000). *Abbreviations*: CI, confidence interval; SMD, standardized mean differences.

The pooled effect is reported in **Fig 5** on moderator assessment type. Based on the 3-level model, there was no discernable effect (SMD = 0.00, 95% CI: -0.21 to 0.21, p = 1.000). Examination of the studentized residuals and Cook’s distance revealed no outliers or potentially influential studies or effects. Inspection of the funnel plot nor the regression test indicated asymmetry (p = 0.174).

According to the Q-test, the true outcome has a low risk of being heterogeneous (Q = 15, *p*<0.165, tau^2^ = 0.036, I^2^ = 29%). The majority of the variance was at level-1 (66%), followed by level-2 (34%). Neither cancer type (p = 0.738), study duration (p = 0.936), participant age (p = 0.888) or exercise prescription type (p = 0.575) were significant effect moderators. However, physical function measurement method was a significant moderator (p = 0.012), decreasing the overall variance by 34%. The subgroup analysis for physical function method is presented in **Fig 5**. There was a small increase for Stand30 (p = 0.036) and a moderate decrease for self-report (p = 0.018). There were trivial-small, non-significant increases for 6MWT (p = 0.093) and Stand1 (p = 0.570), and negligible-small, non-significant decreases for grip strength (p = 0.729) and TUG (p = 0.243).

#### 3.3.5 Fatigue

In total of *k* = 14 studies were included, from which *u* = 18 effect sizes were extracted with a total sample of n = 407. The adjudicated mean quality of the articles was 2 (out of 3), with a range of 1 to 3. The observed SMD ranged from –7.3 to 0.5, with the majority of estimates (67%) being negative (beneficial), such that a smaller number represents less fatigue.

**Fig 6:**
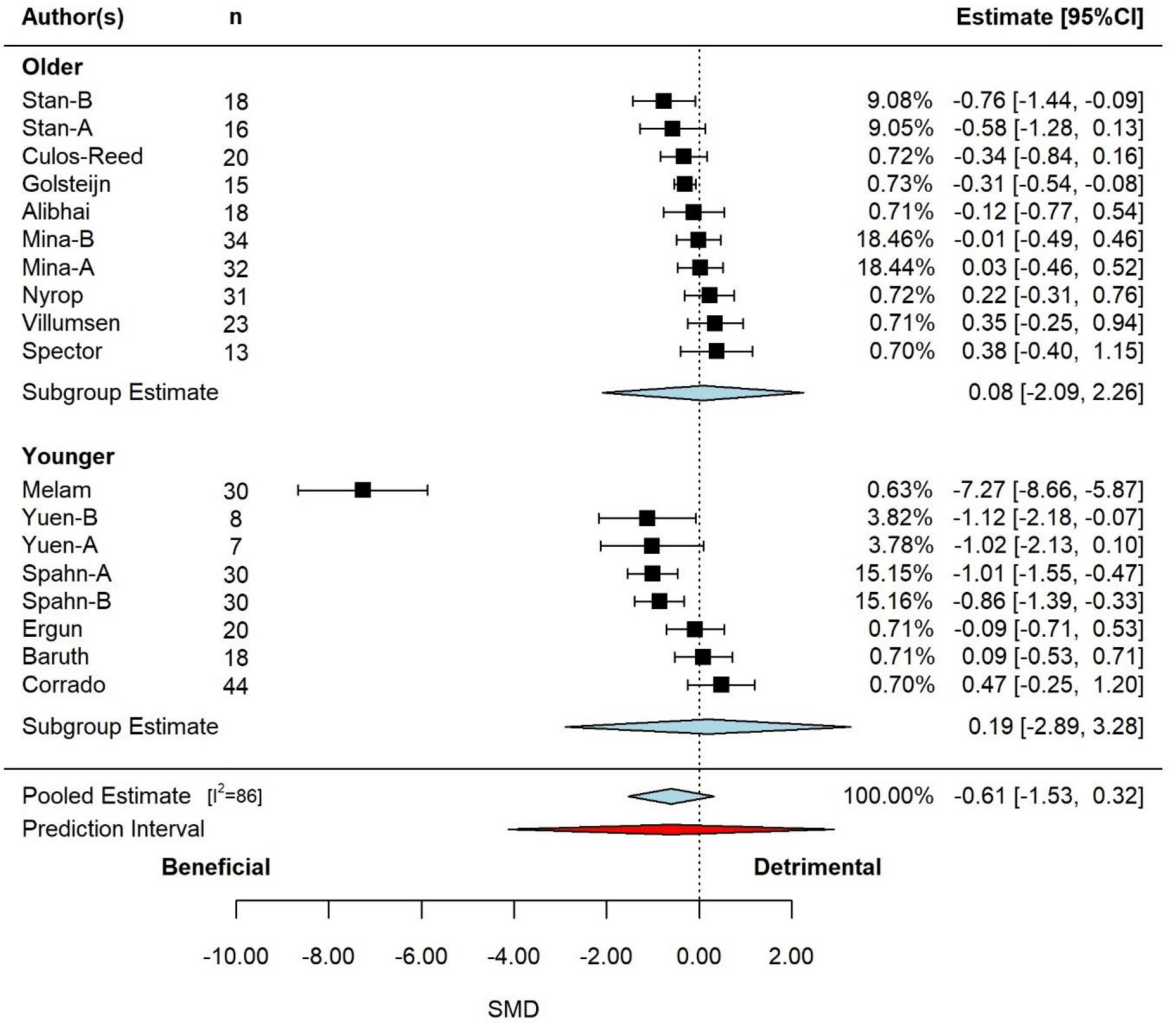
Pooled effect of exercise intervention on fatigue, with sub-group analysis by age (k = 7, u = 12, n = 285). Based on the 3-level model, there was a moderate but non-significant effect (SMD = -0.61, 95% CI: -1.53 to 0.32, p *=* 0.198). *Abbreviations*: CI, confidence interval; SMD, standardized mean differences.

The pooled effect is reported in **Fig 6** on moderator age. Based on the 3-level model, there was a moderate but non-significant effect (SMD = -0.61, 95% CI: -1.53 to 0.32, p *=* 0.198). Examination of the studentized residuals and Cook’s distance revealed that one study (Melam [71]) may be an outlier and could be considered influential. Removal of Malem decreased the size of the estimated effect (SMD = -0.18, 95% CI: -0.44 to 0.07, p *=* 0.163). Inspection of the funnel and the regression test likely indicated funnel plot asymmetry (p<0.001).

According to the Q-test, the true outcomes appear to be heterogeneous (Q = 132, p<0.001, tau^2^ = 0.55, I^2^ = 87%). The majority of the variance was at level-3 (97%), with the remainder of the variance at level-1 (3%). Neither cancer fatigue measurement method (p = 0.913), cancer type (p = 0.410), study duration (p = 0.432), participant age (p = 0.167) or exercise prescription type (p = 0.894) were significant effect moderators.

## 4. Discussion

The aim of this meta-analysis was to investigate the effects of home-based exercise interventions on BCa and PCa survivors. The main findings were that home-based exercise leads to a small, but significant (SMD = 0.30, p = 0.042) increase in QoL. Additionally, small increases were found in PA (SMD = 0.49, p<0.001) and CRF (SMD = 0.45, p = 0.056). Home-based exercise did not affect physical function SMD = 0.00, p = 0.100) and there were moderate but non-significant (SMD = -0.61, p = 0.198) improvements reported in fatigue.

### 4.1 Limitations

Several limitations should be borne in mind when considering the findings of this meta-analysis. First, the sample size of included trials was generally small, and a limited number of trials reported all variables of interest. Second, the small number of trials limits the conclusions drawn from the sub-group analysis; further trials are required to delineate the interactions more clearly between home-based exercise interventions. Third, the quality of the included trials was generally fair (median quality score of 2 and a mean score of 2.1 on a scale of 1 (poor) to 3 (high) quality) and lacked information on intervention adherence and compliance.

### 4.2 Quality of life

Our findings suggest that home-based exercise results in a small but improvement in QoL regardless of the exercise type, intervention duration, age, or cancer (BCa or PCa) type. These data expand on the recent systematic review [20] which established that home-based exercise is feasible in a variety of cancer survivors. Arguably any improvement in QoL is meaningful as enhanced QoL is critical in survivorship. Interestingly, it appears that the questionnaire used may impact the magnitude of QoL quantified. EORTC may be more sensitive to physical changes and FACT may be more sensitive to psychosocial changes [81]. For example, every study that assessed QoL using the EORTC reported some degree of improvement (positive or beneficial change) whereas studies that assessed QoL using the FACT reported mixed findings. Investigators utilizing exercise training to improve QoL may wish to consider EORTC as it may better detect physical changes initiated by PA. Both the EORTC and FACT are valid and reliable questionnaires, but investigators should consider the purpose of their evaluation (physical or psychosocial) when selecting which measurement tool to use [81,82].

These data suggest that home-based exercise improves physical outcomes (function, CRF) contributing to an enhanced QoL but may not improve psychosocial outcomes [83]. Since home-based exercise is completed without supervision or a community it may not confer the same social benefit as supervised or group exercise training [84–86]. There is a significant positive association between social support and PA [87]. In wake of the COVID-19 pandemic and a shift to the virtual delivery of exercise programs [84], we must consider ways to increase social support for cancer survivors. Furthermore, qualitative data investigating psychosocial benefits from PA in breast and prostate cancer survivors suggest that despite facing many barriers to PA engagement, support from other cancer survivors or family/friends should be considered in exercise prescription [88,89].

Our hypothesis that home-based exercise training improves QoL in BCa and PCa survivors was confirmed which is meaningful for survivors, especially those living in rural communities. There was no asymmetry, and the data was heterogenous. When comparing these data to meta-analyses investigating supervised exercise training, we see improvements in QoL of similar magnitude. QoL increased following supervised exercise training in two BCa (SMD = 0.35; 95% CI: 0.15–0.54 [90] and SMD = 0.45, 95% CI 0.20–0.69 [91]) and one PCa (SMD = 0.29; 95% CI = 0.10–0.49 [5]) meta-analysis. Home-based exercise in this study resulted in a highly comparable significant increase in QoL (SMD = 0.30, 95% CI: 0.01 to 0.60). Therefore, unsupervised home-based exercise training is an advantageous strategy for BCa/PCa survivors since it requires far less resources (e.g., supervised exercise facility, commuting time, etc.) than supervised exercise training and results in comparable increases in QoL so long as the goals set are reasonable [92].

When comparing our findings to supervised exercise training, improvements in QoL between the training strategies is of similar magnitude. Supervised exercise training increased QoL in two BCa (SMD = 0.35; 95% CI: 0.15–0.54 [90] and SMD = 0.45, 95% CI 0.20–0.69 [91]) and one PCa (SMD = 0.29; 95% CI = 0.10–0.49 [5]) meta-analyses, which is comparable to increase in QoL shown in the current study using home-based exercise (SMD = 0.30, 95% CI: 0.01 to 0.60). Additionally, although the studies included in this investigation explored different types of exercise [i.e., aerobic exercise only, resistance exercise only, aerobic and resistance training combined, walking, or other (yoga, exergaming, or gardening)] the exercise prescription type did not significantly impact the results. Therefore, for older cancer survivors the type of exercise prescribed should be based on feasibility and potential enjoyment. Further, the majority of studies (N = 15) prescribed an exercise frequency in line with the current American College of Sports Medicine (ACSM) guidelines of achieving 150 minutes of moderate-to-vigorous physical activity on 3–5 days per week. Therefore, unsupervised home-based exercise training is an advantageous strategy for BCa/PCa survivors since it requires far less resources (e.g., supervised exercise facility, commuting time, etc.) than supervised exercise training and results in highly comparable increases in QoL.

### 4.3 Physical activity, cardiorespiratory fitness, physical function, and fatigue

Secondary aims of this meta-analysis were to investigate the effect of home-based exercise training on PA, CRF, physical function, and fatigue. Engaging in PA throughout the day is important in mitigating sedentary behavior and engaging in healthy lifestyle behaviors to prevent chronic disease risk. Due to a high prevalence of cardiometabolic disease in BCa/PCa survivors [93,94], PA engagement is very important to survivorship as it contributes to CRF and helps prevent secondary disease [13,14]. Overall, there is a moderate improvement (SMD = 0.49, p<0.001) in PA reported following home-based exercise training. Our hypothesis that home-based exercise would improve PA was confirmed. However, despite no asymmetry our data had moderate heterogeneity. The heterogeneity could be due to variation in baseline PA levels between studies or variation in PA assessment type (self-report vs. accelerometry). In this meta-analysis, approximately half of the included studies reported BCa/PCa survivors that were physically active [64,67,69,72,77] (meeting ACSM guidelines of 150 minutes of moderate intensity PA per week) at baseline. It is also important to note that only 3 studies objectively measured PA using accelerometry [69,75,80], with the majority of studies using self-reported PA measurements. Studies that utilized accelerometry reported small improvements in PA (SMD = 0.28), whereas self-reported PA was reported to be larger (SMD = 0.54). This suggesting that BCa/PCa survivors over-estimate their physical activity levels. Investigators should keep this in mind when designing future interventions and use accelerometry to measure PA whenever possible to ensure objective accurate measurements [95].

Following home-based exercise training there were also small improvements in CRF that approached significance (SMD = 0.49, p = 0.056). The data had asymmetry likely due to one outlier and considerable heterogeneity which could also be due to variance in baseline CRF similar to PA. Aerobic exercise training resulted in the largest (SMD = 1.27) improvements in CRF, followed by strength training (SMD = 0.89) and then combined (SMD = 0.28). Both aerobic and strength training can benefit CRF, so the participants goals should be considered when designing exercise prescription.

Interestingly physical function did not improve following home-based exercise training (SMD = 0.00, p = 1.00) with no asymmetry or heterogeneity. Physical function is typically considered a key determinant in predicting QoL [16,96]. For example, if someone lacks physical function, then they likely also have a reduced QoL. Therefore, we were surprised that QoL increased despite no improvement in physical function. We postulate that perhaps physical function does not seem to improve because i) we do not have a universal means of assessment in the field or across the studies included in this meta-analysis; or ii) upon further examination of the participants in the study physical function was relatively average at baseline compared to older adults without BCa/PCa. Grip strength and 6-minute walk reported were average for participants and timed stand tests/TUG were just below average indicating that the participants in this meta-analysis may have not had the expected physical function determinants usually expected in BCa/PCa survivors.

Fatigue is one of the most common side effects for BCa/PCa survivors, yet we found only a moderate, non-significant (SMD = -0.61, p = 0.198) decrease in fatigue with asymmetry likely from one outlier and heterogeneity in the data. However, only three studies included in this meta-analysis reported severe fatigue at baseline [65,66,73]. The remaining studies reported only moderate or mild fatigue. The variation in baseline levels of fatigue may be why there was heterogeneity and non-significant findings. Furthermore, the result of exercise training on fatigue was highly varied. A meta-analysis of the effects of supervised exercise on fatigue in BCa found a clear significant improvement in fatigue after both aerobic (SMD = -0.51) and resistance training (SMD = -0.41) [97]. Therefore, we hypothesized that home-based exercise would elicit similar findings. At first, we were surprised by the non-significant improvement in fatigue, especially considering the large effect. However, upon further investigation, the majority of BCa/PCa survivors included in this meta-analysis do no report severe fatigue at baseline. Currently undergoing chemotherapy or radiation was an exclusion criterion which may explain why participants were not suffering from severe fatigue at baseline. Since many of the participants included in this study were not suffering from severe fatigue at baseline is it not surprising that exercise training did not improve fatigue since it was not actually a problem at baseline. Additionally, there is no standardized procedure for assessing fatigue which could explain some of the variation in these data. Further research is needed to design a robust tool to assess fatigue.

## 5. Conclusions

Cancer survivorship is a dynamic process which evolves over time for BCa/PCa survivors. Health outcomes such as QoL, PA, CRF, physical function, and fatigue contribute towards the life of a BCa/PCa survivor after cancer. Unsupervised, home-based exercise training may be a good alternative for survivors who do not have access or interest in participating in supervised or group-based programs (Table 2). However, we must consider limitations of the current literature (e.g., limited number of high-quality trials, small sample sizes) along with the long-term sustainability of this approach [84]. Engaging in home-based exercise results in small improvements in QoL, which is arguably the most important outcome investigated by this current study because it is critical in survivorship. Therefore, clinicians and caregivers should encourage BCa/PCa survivors to engage in exercise to improve QoL, PA and CRF. Future research is needed to continue refining exercise prescription/guidelines for BCa/PCa survivors and additional research is needed to effectively target the needs of various cancer types and exercise delivery to other timepoints among the cancer care continuum.

**Table 2:** Summary of implications found including current state of knowledge, new findings, and future directions of study.

**What did we know prior to this study?** • Supervised or group-based exercise training is beneficial for BCa/PCa survivors • Improving QoL, PA, CRF, physical function, and fatigue are important components of survivorship. • Not all BCa/PCa survivors have access to exercise training such as those living in rural communities • COVID-19 has emphasized barriers to supervised exercise.
**What didn’t we know prior to this study?** • Does home-based, unsupervised exercise training improve QoL, and subsequent outcomes (physical activity, cardiorespiratory fitness, physical function and fatigue) in BCa/PCa survivors? • Is home-based exercise a feasible alternative to supervised exercise training to improve QoL? • How do we compare between study findings due to variation in study procedures and assessment types?
**What does this study add?** • Home-based exercise training improves QoL and may also improve PA and CRF. • Home-based unsupervised exercise results in similar increases in QoL when compared to supervised exercise meta-analytic findings indicating it may be an advantageous and less resource intensive alternative. • 3-Level meta-analysis methodology allows for between study comparison despite no standardize approach to exercise prescription for BCa/PCa survivors.
**How do we use this new information?** • Engaging in home-based exercise improves QoL in BCa/PCa survivors. Clinicians and caregivers should encourage BCa/PCa survivors to engage in exercise. • Exercise training can successfully increase QoL without supervision which is encouraging for survivors living in rural communities or without access to supervised training • Policy makers such encourage promotion of home-based exercise and disseminate educational material to increase knowledge of exercise training benefits at home.

## Supporting information

S1 Fig Funnel plot of observed outcomes (standardized mean difference) for quality of life. **Description.** Funnel plot constructed using 3-level model. Includes *k* = 17 studies, *u* = 23 effect sizes, and total sample of n = 657. Regression intercept test constructed by specifying the standard error of the observed outcomes as a moderator in a 3-level model. **Interpretation.** No asymmetry (p = 0.174) was revealed following inspection of the funnel plot and regression test.
(DOCX)

S2 Fig Funnel plot of observed outcomes (standardized mean difference) for physical activity. **Description.** Funnel plot constructed using 3-level model. Includes *k* = 10 studies, *u* = 14 effect sizes, and total sample of n = 630. Regression intercept test constructed by specifying the standard error of the observed outcomes as a moderator in a 3-level model. **Interpretation.** Inspection of the funnel plot nor the regression test indicated asymmetry (p = 1.000).
(DOCX)

S3 Fig Funnel plot of observed outcomes (standardized mean difference) for cardiorespiratory fitness. **Description.** Funnel plot constructed using 3-level model. Includes *k* = 7 studies, *u* = 14 effect sizes, and total sample of n = 312. Regression intercept test constructed by specifying the standard error of the observed outcomes as a moderator in a 3-level model. **Interpretation.** Inspection of the funnel plot and the regression test indicated likely asymmetry (p = 0.067).
(DOCX)

S4 Fig Funnel plot of observed outcomes (standardized mean difference) for physical function. **Description.** Funnel plot constructed using 3-level model. Includes *k* = 7, studies, *u* = 12, effect sizes, and total sample of n = 285. Regression intercept test constructed by specifying the standard error of the observed outcomes as a moderator in a 3-level model. **Interpretation.** Inspection of the funnel plot nor the regression test indicated asymmetry (p = 0.174).
(DOCX)

S5 Fig Funnel plot of observed outcomes (standardized mean difference) for fatigue. **Description.** Funnel plot constructed using 3-level model. Includes *k* = 14, studies, *u* = 18 effect sizes, and total sample of n = 407. Regression intercept test constructed by specifying the standard error of the observed outcomes as a moderator in a 3-level model. **Interpretation.** Inspection of the funnel and the regression test likely indicated funnel plot asymmetry (p< 0.001).
(DOCX)

S1 Table PRISMA 2020 abstracts checklist. (DOCX)

S2 Table PRISMA 2020 checklist. (DOCX)
