# Supplementary material for: Home-based exercise improves quality of life in breast and prostate cancer survivors: A meta-analysis
Source: PLoS One. 2023 Apr 20;18(4):e0284427. doi: 10.1371/journal.pone.0284427 (PMC10118157; doi:10.1371/journal.pone.0284427)
Supplement: S1 Fig — Description. Funnel plot constructed using 3-level model. Includes k = 17 studies, u = 23 effect sizes, and total sample of n = 657. Regression intercept test constructed by specifying the standard error of the observed outcomes as a moderator in a 3-level model. Interpretation. No asymmetry (p = 0.174) was revealed following inspection of the funnel plot and regression test. (DOCX) [file pone.0284427.s001.docx]

# FIGURE S1.

## **Title:** Funnel plot of observed outcomes (standardized mean difference) for quality of life.


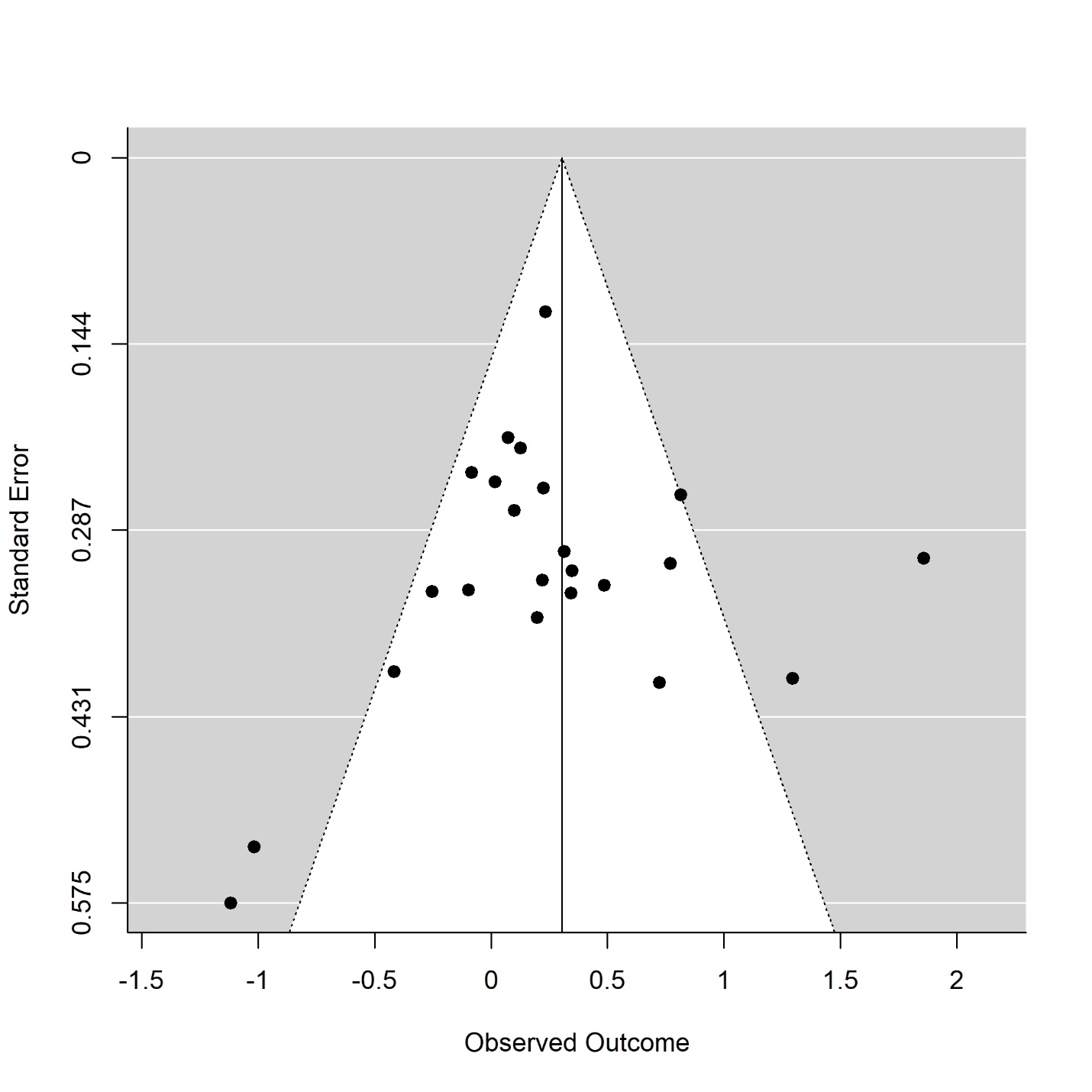


**Description.** Funnel plot constructed using 3-level model. Includes *k*=17 studies, *u* = 23 effect sizes, and total sample of n = 657. Regression intercept test constructed by specifying the standard error of the observed outcomes as a moderator in a 3-level model.

**Interpretation.** No asymmetry (p=0.174) was revealed following inspection of the funnel plot and regression test.
