# Supplementary material for: Home-based exercise improves quality of life in breast and prostate cancer survivors: A meta-analysis
Source: PLoS One. 2023 Apr 20;18(4):e0284427. doi: 10.1371/journal.pone.0284427 (PMC10118157; doi:10.1371/journal.pone.0284427)
Supplement: S2 Fig — Description. Funnel plot constructed using 3-level model. Includes k = 10 studies, u = 14 effect sizes, and total sample of n = 630. Regression intercept test constructed by specifying the standard error of the observed outcomes as a moderator in a 3-level model. Interpretation. Inspection of the funnel plot nor the regression test indicated asymmetry (p = 1.000). (DOCX) [file pone.0284427.s002.docx]

# FIGURE S2.

## **Title:** Funnel plot of observed outcomes (standardized mean difference) for physical activity.


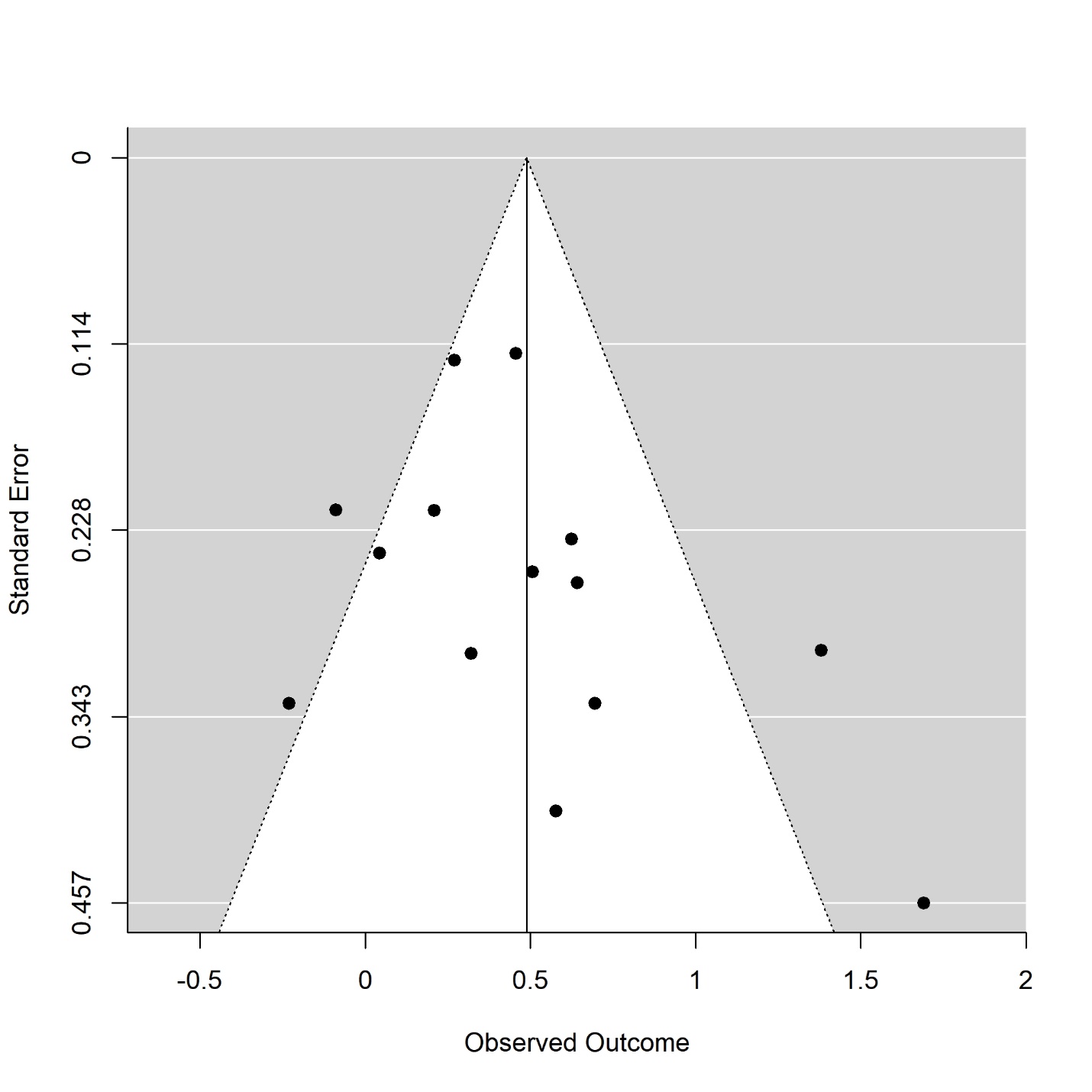


**Description.** Funnel plot constructed using 3-level model. Includes *k*=10 studies, *u* = 14 effect sizes, and total sample of n = 630. Regression intercept test constructed by specifying the standard error of the observed outcomes as a moderator in a 3-level model.

**Interpretation.** Inspection of the funnel plot nor the regression test indicated asymmetry (p=1.000).
