# Supplementary material for: Home-based exercise improves quality of life in breast and prostate cancer survivors: A meta-analysis
Source: PLoS One. 2023 Apr 20;18(4):e0284427. doi: 10.1371/journal.pone.0284427 (PMC10118157; doi:10.1371/journal.pone.0284427)
Supplement: S4 Fig — Description. Funnel plot constructed using 3-level model. Includes k = 7, studies, u = 12, effect sizes, and total sample of n = 285. Regression intercept test constructed by specifying the standard error of the observed outcomes as a moderator in a 3-level model. Interpretation. Inspection of the funnel plot nor the regression test indicated asymmetry (p = 0.174). (DOCX) [file pone.0284427.s004.docx]

# FIGURE S4.

## **Title:** Funnel plot of observed outcomes (standardized mean difference) for physical function.


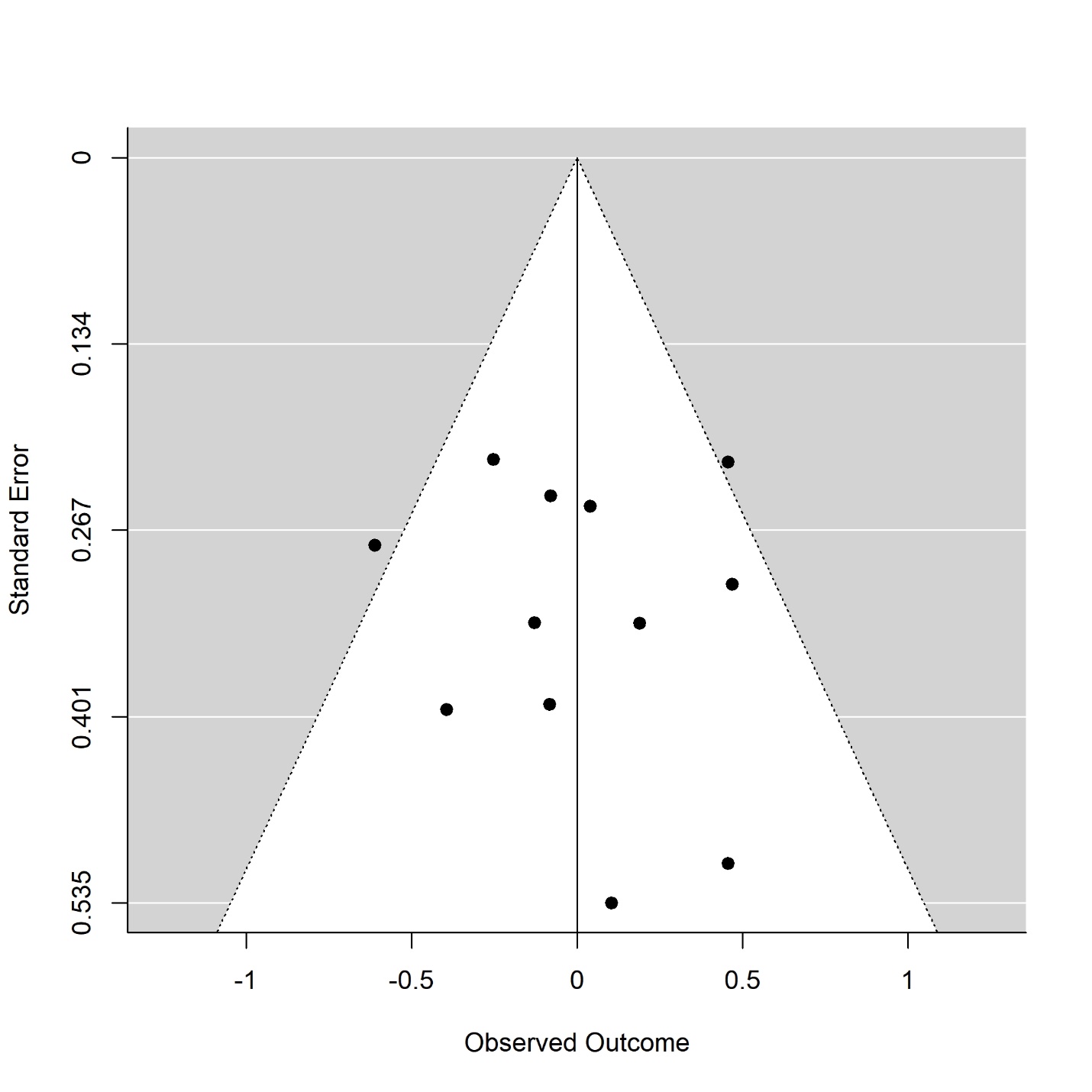
**Description.** Funnel plot constructed using 3-level model. Includes *k*=7, studies, *u* = 12, effect sizes, and total sample of n = 285. Regression intercept test constructed by specifying the standard error of the observed outcomes as a moderator in a 3-level model.

**Interpretation.** Inspection of the funnel plot nor the regression test indicated asymmetry (p=0.174).
