# Supplementary material for: Home-based exercise improves quality of life in breast and prostate cancer survivors: A meta-analysis
Source: PLoS One. 2023 Apr 20;18(4):e0284427. doi: 10.1371/journal.pone.0284427 (PMC10118157; doi:10.1371/journal.pone.0284427)
Supplement: S5 Fig — Description. Funnel plot constructed using 3-level model. Includes k = 14, studies, u = 18 effect sizes, and total sample of n = 407. Regression intercept test constructed by specifying the standard error of the observed outcomes as a moderator in a 3-level model. Interpretation. Inspection of the funnel and the regression test likely indicated funnel plot asymmetry (p< 0.001). (DOCX) [file pone.0284427.s005.docx]

# FIGURE S5.

## **Title:** Funnel plot of observed outcomes (standardized mean difference) for fatigue.


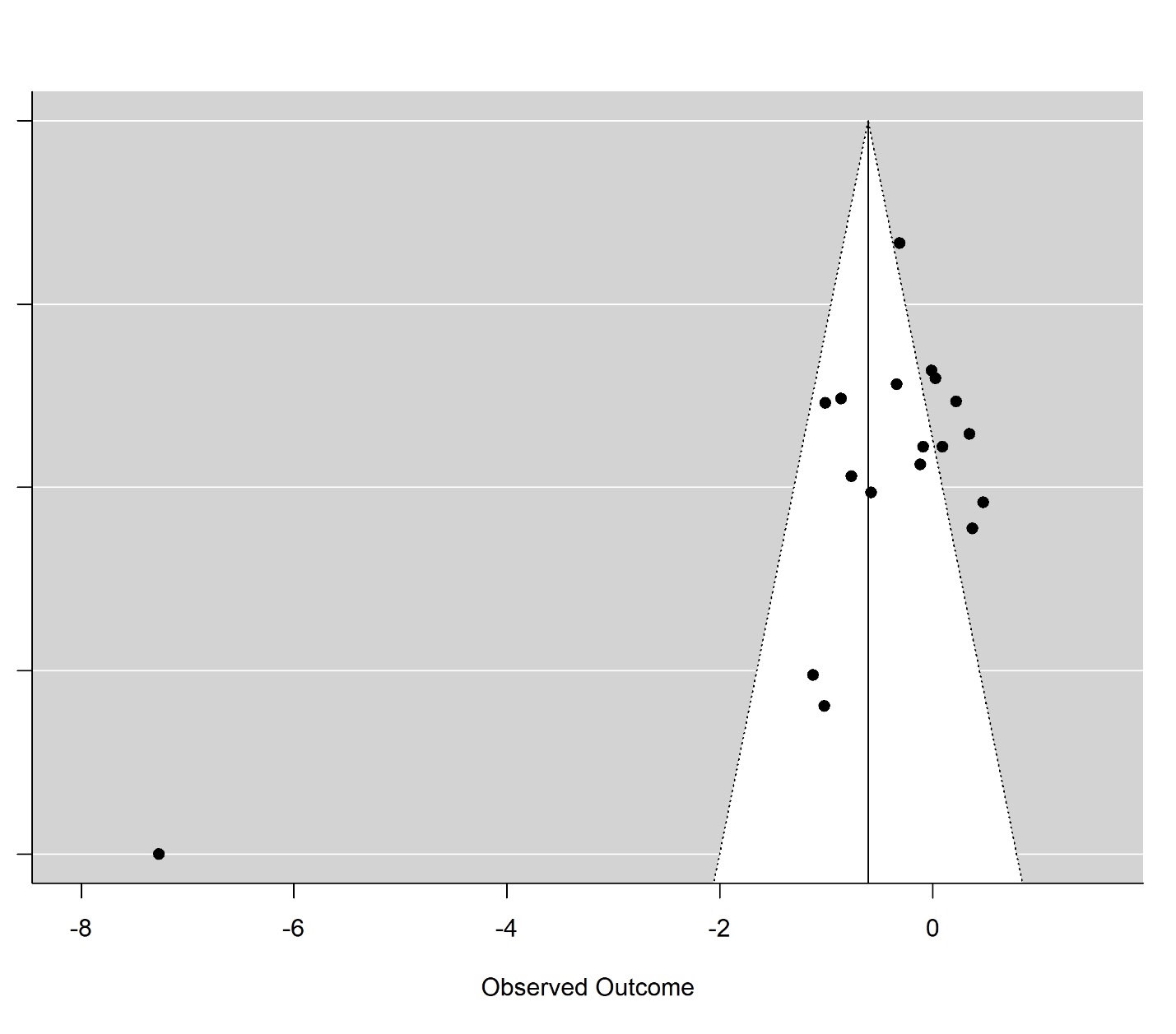
**Description.** Funnel plot constructed using 3-level model. Includes *k*=14, studies, *u* = 18 effect sizes, and total sample of n = 407. Regression intercept test constructed by specifying the standard error of the observed outcomes as a moderator in a 3-level model.

**Interpretation.** Inspection of the funnel and the regression test likely indicated funnel plot asymmetry (p< 0.001).
